# A Self-Guided Internet-Based Intervention for the Reduction of Gambling Symptoms: A Randomized Clinical Trial

**DOI:** 10.1001/jamanetworkopen.2024.17282

**Published:** 2024-06-21

**Authors:** Lara Rolvien, Lisa Buddeberg, Josefine Gehlenborg, Swantje Borsutzky, Steffen Moritz

**Affiliations:** 1 Department of Psychiatry and Psychotherapy, University Medical Center Hamburg-Eppendorf, Hamburg, Germany

## Abstract

**Importance:**

Most individuals with problem gambling or gambling disorder remain untreated due to barriers to treatment. Limited research exists on alternative treatments.

**Objective:**

To investigate the efficacy of a self-guided internet-based intervention for individuals with gambling problems and to identify potential outcome moderators.

**Design, Setting, and Participants:**

This single-center randomized clinical trial was conducted from July 13, 2021, to December 31, 2022, at the University Medical Center Hamburg-Eppendorf. Participants were recruited across Germany for 2 assessments (before intervention [t_0_] and 6 weeks after intervention [t_1_]). Eligible participants were individuals aged 18 to 75 years with gambling problems, internet access, German proficiency, and willingness to participate in 2 online assessments.

**Intervention:**

The self-guided internet-based intervention was based on cognitive behavioral therapy, metacognitive training, acceptance and commitment therapy, and motivational interviewing.

**Main Outcome and Measures:**

The primary outcome was change in gambling-related thoughts and behavior as measured with the pathological gambling adaption of the Yale-Brown Obsessive-Compulsive Scale. Secondary outcomes were change in depressive symptoms, gambling severity, gambling-specific dysfunctional thoughts, attitudes toward online interventions, treatment expectations, and patient satisfaction.

**Results:**

A total of 243 participants (154 [63.4%] male; mean [SD] age, 34.73 [10.33] years) were randomized to an intervention group (n = 119) that gained access to a self-guided internet-based intervention during 6 weeks or a wait-listed control group (n = 124). Completion at t_1_ was high (191 [78.6%]). Results showed a significantly greater reduction in gambling-related thoughts and behavior (mean difference, −3.35; 95% CI, −4.79 to −1.91; *P* < .001; Cohen *d* = 0.59), depressive symptoms (mean difference, −1.05; 95% CI, −1.87 to −0.22; *P* = .01; Cohen *d* = 0.33), and gambling severity (mean difference, −1.46; 95% CI, −2.37 to −0.54; *P* = .002; Cohen *d* = 0.40) but not in gambling-specific dysfunctional thoughts (mean difference, −1.62; 95% CI, −3.40 to 0.15; *P* = .07; Cohen *d* = 0.23) favoring the intervention group. Individuals in the intervention group who had a positive treatment expectation and more severe gambling-specific dysfunctional thoughts and gambling symptoms benefited more on the primary outcome relative to the control group.

**Conclusions and Relevance:**

In this randomized clinical trial, the effectiveness of a self-guided internet-based intervention for individuals with self-reported problematic gambling behavior was demonstrated when measured 6 weeks after start of the intervention. The study’s findings are particularly relevant given the increasing need for accessible and scalable solutions to address problematic gambling.

**Trial Registration:**

bfarm.de Identifier: DRKS00024840

## Key Points

**Question:**

Is a self-guided internet-based intervention for individuals with gambling symptoms effective in reducing problem gambling thoughts and behavior, comorbid depressive symptoms, and gambling-related thought distortions, and what factors moderate its effectiveness?

**Findings:**

In this randomized clinical trial of 243 participants with self-reported gambling problems, the intervention group who received access to the program during a 6-week period improved significantly in gambling symptoms and depressive symptoms but not in gambling-specific thought distortions after treatment. Treatment expectation and symptom severity were moderating factors.

**Meaning:**

These findings suggest that online self-help may be used effectively in the treatment of problem gambling symptoms.

## Teaser

This randomized clinical trial of individuals with gambling problems assesses the effectiveness of a self-guided internet-based intervention for the reduction of gambling-related thoughts and behavior.

## Introduction

Pathological gambling, now classified as a behavioral addiction in the *Diagnostic and Statistical Manual of Mental Disorders* (Fifth Edition; *DSM*-5),^1^ poses significant health risks beyond finances, including psychosocial, physical, and psychological impacts.^2^ Despite available treatments, fewer than 10% of individuals with a gambling disorder seek treatment, leading to a large treatment gap.^3^ The most common treatment barriers are the desire to deal with the problem alone, shame and stigma, reluctance to acknowledge the problem, lack of knowledge regarding treatment options, problems in seeking treatment, high cost of treatment, and a dearth of gambling-specific treatment services.^4,5^ Addressing these barriers requires low-threshold, evidence-based psychotherapeutic treatment programs.

Internet-based treatments offer a promising solution for the treatment gap and have been proven effective for psychological disorders, such as anxiety disorders and depression.^6,7^ These interventions can be self-guided (no additional professional help), guided (with additional professional help^8^), or blended (combining an internet-based intervention with classic face-to-face treatment),^9^ with higher effect sizes observed for those with professional support.^10^ Although evidence supports their effectiveness for problematic and pathological gambling behavior (for a current review, see Rodda^11^), studies show mixed results, often referencing studies with inactive control groups.^12,13,14^ Only 1 meta-analysis assessed internet-based interventions for gambling problems across 13 studies.^15^ It found a medium effect size (Hedges *g* = 0.73) for general gambling symptom improvement after treatment but noted significant heterogeneity. Studies without a control group (n = 4) yielded larger effects (Hedges *g* = 1.23) compared with those with a control group (n = 9; Hedges *g = *0.47). Therapist-guided interventions had larger effect sizes (Hedges *g* = 1.23) than unguided ones (Hedges *g* = 0.39), and some studies had very small samples.^16,17^

Given the low rates of treatment and high barriers, assessing attitudes toward online interventions and treatment expectations is crucial. However, to our knowledge, no study has evaluated attitudes toward online interventions in this group. Limited data on therapy expectancy show relatively high treatment expectations (76.9% on the Credibility/Expectancy Questionnaire) in individuals with a gambling disorder before starting internet-based treatment.^18^

More well-conducted randomized clinical trials with larger samples are needed to assess self-guided internet-based interventions for gambling problems. A previous randomized clinical trial^12^ examined a self-guided internet-based intervention for individuals with gambling problems developed by our working group but could not find significant effects, possibly due to the small sample size (n = 150). Since then, we have enhanced the program to increase treatment motivation and engagement with the program (see the Intervention section).

This study aimed to (1) assess the effect of the improved and expanded version of this online intervention on gambling-related thoughts and behavior (primary outcome), depressive symptoms, gambling-specific dysfunctional thoughts, and gambling severity (secondary outcomes) and (2) to identify any variables that might moderate the effect on the primary outcome. We expected the intervention group to show significantly greater reductions in symptoms compared with the wait-listed control group.

## Methods

### Study Design

This randomized clinical trial included an intervention group that used the self-guided internet-based intervention Restart (Neustart)^19^ for 6 weeks and a wait-listed control group that accessed it after completing the postassessment. Both groups had access to treatment as usual during the study period. The local ethics committee of the University Medical Center Hamburg-Eppendorf approved the trial protocol and statistical analysis plan (Supplement 1), and the study was conducted in accordance with the Declaration of Helsinki.^20^ Participants were required to provide written informed consent during the baseline assessment. The intervention program uses secure encryption. The study was registered with the German Clinical Trials Register, conducted at the University Medical Center Hamburg-Eppendorf, and reported following the Consolidated Standards of Reporting Trials (CONSORT) reporting guidelines.^21^

### Participants

Participants were recruited via social media channels (eg, Facebook groups and Instagram) and small flyers placed in gambling halls across Germany. Inclusion criteria were (1) subjective need for help because of gambling problems, (2) internet access, (3) age between 18 and 75 years, (4) sufficient command of the German language, and (5) willingness to participate in 2 online assessments. A self-reported lifetime diagnosis of schizophrenia or psychosis or bipolar disorder or mania and suicidal ideation were exclusion criteria (suicidal ideation was assessed with the last item of the Patient Health Questionnaire [PHQ-9]; contact information for help hotlines and facilities was provided if the cutoff of 2 points was exceeded). Data on race and ethnicity were not collected because they were not relevant to the study’s research question.

### Randomization

Participants were randomized after providing informed consent and completing the baseline assessment. The first participant was included on July 13, 2021, and the last on December 31, 2022. Randomization was performed automatically via the online survey program and was therefore concealed from the study’s administrators. The random allocation ratio was parallel (1:1) and was fully automated by Enterprise Feedback Suite (EFS) Survey, version 22.1 (Tivian XI GmbH). Due to the study setup (online surveys and self-assessment only), the study was unmasked. Participants accessed the baseline assessment via the study website or recruitment posts on social media (eg, Instagram).

### Procedures

Before intervention (t_0_) and 6 weeks after intervention (t_1_), a pseudonymous online assessment via the survey software EFS Survey was conducted. At t_0_, sociodemographic data, psychopathology, inclusion and exclusion criteria, and attitudes (Attitudes Towards Psychological Online Interventions [APOI]) and expectations (Patient Questionnaire on Therapy Expectation and Evaluation [PATHEV]) regarding online interventions were assessed. At t_1_, psychopathology and acceptance were assessed. After completion of t_1_, participants in both groups received a €20 voucher. Participants in the control group then also received access to the online program. Participants were automatically invited to the postassessment via trigger emails. The intervention group received weekly reminders and module presentations. No personal data except for a pseudonymous email address were collected (instructions on how to create a pseudonymous email address were provided). Email addresses were recoded for anonymity, and personalized links ensured correct data matching between assessments.

### Intervention

The internet-based self-help intervention is composed of 12 modules addressing problematic and pathological gambling behaviors and associated emotional symptoms.^22^ The intervention integrates cognitive behavioral, mindfulness, and metacognitive strategies through text, video, and audio content. The modules are interactive, and users are advised to spend 30 to 60 minutes per module and to complete 2 modules weekly (module order can be freely chosen). It operates as a self-guided intervention with technical support available but not therapeutic support. Login duration is monitored via program log files.

Since the last study^12^ on the program, it has been enhanced based on earlier feedback. New additions include a sports-betting module and an initial motivational module (based on motivational interviewing techniques^23^). Four characters with gambling problems personalize the treatment, appearing in short case stories that are supplemented with illustrations. Most importantly, participants are encouraged to use the self-help smartphone application Cogito^24^ (for studies on earlier versions of this application as well as a detailed description of the app content, see Bruhns et al^25,26^) alongside the intervention program. The application offers self-help exercises based on cognitive behavioral therapy,^27^ metacognitive training,^28^ and third-wave techniques, such as mindfulness and acceptance,^29^ primarily for depression. Additionally, the application contains exercises that specifically target gambling symptoms. The application has been improved with a new design, images, and content since the earlier studies. The application was promoted in the first email giving login data and was featured prominently in the online program’s main menu.

### Outcomes

#### PG-YBOCS Scores

The pathological gambling adaptation of the Yale-Brown Obsessive-Compulsive Scale (PG-YBOCS)^30^ served as the primary outcome and consists of 2 subscales, each with 10 items assessing gambling severity during the previous week. The first subscale focuses on gambling-related thoughts and urges and the second on gambling-related behavior. With a total score ranging from 0 to 40, scores of 0 to 7 indicate subclinical, 8 to 15 mild, 16 to 23 moderate, 24 to 31 severe, and 32 to 40 extreme symptom severity. The PG-YBOCS showed an excellent internal consistency of Cronbach α = 0.94 in the current study.

#### PHQ-9 Scores

The Patient Health Questionnaire Module 9 (PHQ-9)^31^ is a self-assessment questionnaire that consists of 9 items to measure symptoms of major depression based on the *DSM-IV*. The 4-point rating scale for each item ranges from not at all to nearly every day, with a total score between 0 and 27. Total scores between 0 and 4 indicate no or minimal depressive symptoms, scores of 5 to 9 mild depressive symptoms, 10 to 14 moderate depressive symptoms, and 15 to 27 severe depressive symptoms. The internal consistency of the scale was Cronbach α = 0.90.

#### SOGS Scores

Although originally based on *DSM-III* criteria for pathological gambling, the South Oaks Gambling Screen (SOGS)^32^ also correlates with *DSM-IV* and *DSM-5* criteria and is a common measure to assess adult problematic gambling. In this study, we used a self-assessment questionnaire consisting of 20 items. Total scores range from 0 to 20, with indications of no gambling problems (scores, 0-2), at-risk gambling (scores, 3-4), and pathological gambling (scores, 5-20). The SOGS internal consistency was moderate, with a Cronbach α = 0.50.

#### GABS Scores

The Gambling Attitudes and Beliefs Survey (GABS)^33^ measures dysfunctional beliefs and attitudes toward gambling. This self-assessment questionnaire consists of 35 items with a 4-point Likert scale ranging from a score of 1 indicating strongly disagree to 4 indicating strongly agree. The internal consistency of the GABS in the current study was Cronbach α = 0.93.

#### APOI Scores

The APOI^34^ is a self-assessment questionnaire to measure attitudes toward online interventions on 4 dimensions: (1) skepticism and risk perception, (2) trust in therapeutic efficacy, (3) perception of deficits in mechanization, and (4) perception of the advantages of anonymity. The 16 items can be answered on a 5-point rating scale ranging from a score of 1 (indicating do not agree at all) to 5 (indicating fully agree), with possible sum scores ranging from 16 to 80. Higher scores are associated with more positive attitudes. The scale’s internal consistency was Cronbach α = 0.72 in the current study.

#### PATHEV Scores

The PATHEV^35^ assesses expectations on therapeutic treatment. Covering the 3 dimensions of hope of improvement, fear of change, and suitability, the self-assessment questionnaire contains 10 items. The items can be answered on a 5-point rating scale ranging from a score of 1 (indicating not correct at all) to 5 (indicating completely correct). Higher scores demonstrate a higher hope of improvement, fear of change, and suitability (range, 11-55). The PATHEV’s internal consistency was Cronbach α = 0.71 in the current study. We additionally assessed treatment expectations with 1 global item: “I expect to feel much worse/somewhat worse/the same/somewhat better/much better after using the intervention.”

#### Subjective Appraisal Rating Scale Score

The Subjective Appraisal Rating Scale^36^ is a self-assessment questionnaire measuring patient satisfaction and was used to assess the subjective appraisal of the program. It is the German version of the Client Satisfaction Questionnaire, which consists of 8 items with a 4-point rating scale. The higher the total score (range, 8-32), the higher the level of satisfaction with the program. The instrument had a good internal consistency with a Cronbach α = 0.89.

### Statistical Analysis

The sample size calculation was performed with G*Power, version 3.1.9.7^37^ and resulted in a sample size of 199 individuals when assuming a small to medium effect size of *f* = 0.20 (based on the results of a previous study^38^), a power of 0.80, and α = .05. Considering the expected dropout rate of 20%, this resulted in a total sample size of 238 participants.

SPSS Statistics, version 27 (IBM Corp) was used for all statistical analyses. As registered, we performed analyses of covariance (baseline values as covariates, group as independent variable, and post scores of the outcomes as dependent variables) for the intention-to-treat sample to analyze group comparisons over time. For intention-to-treat analyses, missing values were estimated using the expectation-maximization algorithm. Effect sizes for within-group differences are expressed in Cohen *d* (with 0.2 indicating small; 0.5, medium; and 0.8, large). Correlative relationships were analyzed with Pearson correlation coefficients. An exploratory moderation analysis was performed using SPSS PROCESS^39^ for the per-protocol sample. The per-protocol sample was defined as “participated in baseline and postassessment and used the intervention at least once.” Changes in the PG-YBOCS scores (primary outcome) served as the dependent variable; sociodemographic characteristics, psychopathology (total scales), and attitudes toward online interventions as well as treatment expectations (single items and total scales) at baseline served as moderators.

## Results

**Figure 1.**
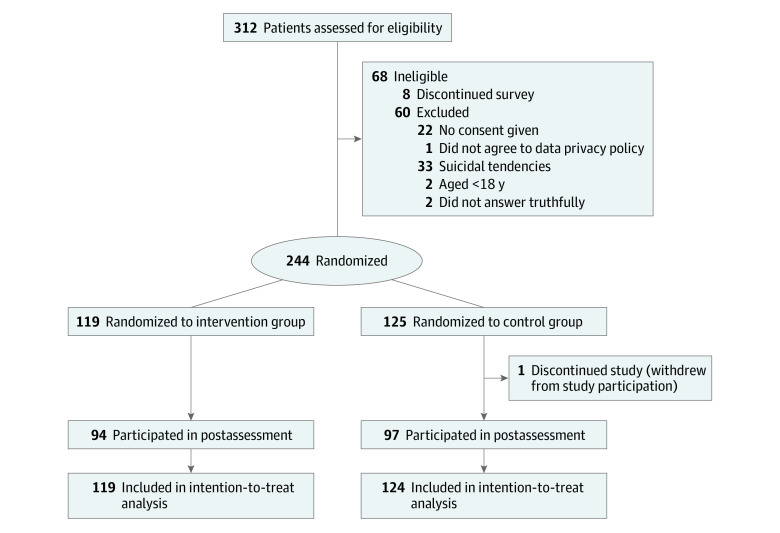
Study Flow Diagram

The total sample included 243 individuals (119 in the intervention group and 124 in the wait-listed control group; 154 [63.4%] male and 89 [36.6%] female; mean [SD] age, 34.73 [10.33] years) (Figure 1). Baseline characteristics and psychopathology data are presented in Table 1. A total of 218 participants (89.7%) were German, 123 (50.6%) were employed full time, and 113 (46.5%) had taken the examination qualifying them for university study. At baseline, the overall sample had a moderate gambling severity (mean [SD] PG-YBOCS score, 17.35 [7.92]), severe gambling-specific dysfunctional thoughts and attitudes (mean [SD] GABS score, 24.65 [10.07]), and mild depressive symptoms (mean [SD] PHQ-9 score, 8.33 [5.67]).

**Table 1.** Baseline Characteristics and Psychopathology (Intention-to-Treat Sample)^a^

Variable	Intervention (n = 119)	Wait-listed control group (n = 124)	Effect size (95% CI)^b^
Demographic characteristics			
Gender			
Male	73 (61.3)	81 (65.3)	0.04 (0.00 to 0.17)
Female	46 (38.7)	43 (34.7)	
Age, mean (SD), y	35.12 (10.86)	34.35 (9.82)	−0.07 (−0.33 to 0.18)
Qualified for university study	60 (50.4)	53 (42.7)	0.13 (0.10 to 0.28)
Nationality			
German	105 (88.2)	113 (91.1)	0.05 (0.00 to 0.18)
Other^c^	14 (11.8)	11 (8.9)	
Professional status (full-time employment)	58 (48.7)	65 (52.4)	0.21 (0.16 to 0.35)
Treatment variables			
Psychotropic medication	7 (5.9)	14 (11.3)	0.10 (0.01 to 0.21)
Current use of self-help	9 (7.6)	4 (3.2)	0.10 (0.00 to 0.21)
Currently in psychotherapy	12 (10.1)	15 (12.1)	0.03 (0.00 to 0.16)
Diagnosed gambling disorder	27 (22.7)	29 (23.4)	0.01 (0.00 to 0.14)
Currently prohibited from gambling	9 (7.6)	12 (9.7)	0.06 (0.02 to 0.21)
Psychopathology scores, mean (SD)			
PG-YBOCS total	16.77 (7.64)	16.71 (7.65)	−0.17 (−0.42 to 0.09)
PG-YBOCS behavior	8.18 (4.08)	8.14 (4.07)	−0.14 (−0.39 to 0.11)
PG-YBOCS thoughts	9.29 (4.25)	8.56 (3.84)	−0.18 (−0.43 to 0.07)
PHQ-9	8.78 (5.66)	8.70 (5.62)	0.14 (−0.12 to 0.39)
GABS	24.05 (10.51)	24.00 (10.54)	−0.13 (−0.38 to 0.12)
SOGS	9.09 (4.67)	9.06 (4.68)	0.00 (−0.25 to 0.25)
Attitudes and expectations scores, mean (SD)			
APOI skepticism	11.37 (3.17)	10.69 (2.77)	−0.23 (−0.48 to 0.02)
APOI trust	14.54 (3.14)	14.02 (3.49)	−0.16 (−0.41 to 0.10)
APOI deficits	12.26 (2.73)	11.89 (3.14)	−0.13 (−0.38 to 0.13)
APOI anonymity	12.32 (2.65)	12.98 (3.07)	0.23 (−0.02 to 0.48)
APOI total	51.23 (7.75)	52.42 (7.45)	0.16 (−0.10 to 0.41)
PATHEV hope	11.31 (3.25)	10.72 (2.64)	−0.20 (−0.45 to 0.05)
PATHEV fear	10.22 (2.74)	10.53 (2.93)	−0.11 (−0.14 to 0.36)
PATHEV suitability	11.91 (2.59)	11.65 (2.17)	−0.11 (−0.36 to 0.14)
PATHEV total	33.44 (5.32)	32.90 (4.53)	−0.11 (−0.36 to 0.14)

Abbreviations: APOI, Attitudes Towards Psychological Online Interventions; GABS, Gambling Attitudes and Beliefs Survey; PATHEV, Patient Questionnaire on Therapy Expectation and Evaluation; PG-YBOCS, pathological gambling adaptation of the Yale-Brown Obsessive Compulsive Scale; PHQ-9, Patient Health Questionnaire–9; SOGS, South Oaks Gambling Screen.

^a^ Data are presented as number (percentage) of study participants unless otherwise indicated.

^b^ Effect size is reported as Cohen *d* along with 95% CIs for all metric variables and as Cramer *V* for all nominal ones. Bootstrapping was performed for 95% CIs for Cramer *V*.

^c^ Other indicates any nationality other than German.

A total of 191 participants (78.6%) completed the postassessment, and the completion rate did not differ between groups (94 [78.9%] in the intervention group and 97 [77.6%] in the control group; Cramer *V* = 0.02; *P* = .88). The mean (SD) number of completed modules was 4.55 (4.98), the mean (SD) number of logins was 2.75 (1.86), and the mean (SD) total use time was 61.22 (63.60) minutes.

### Primary Analyses

**Figure 2.**
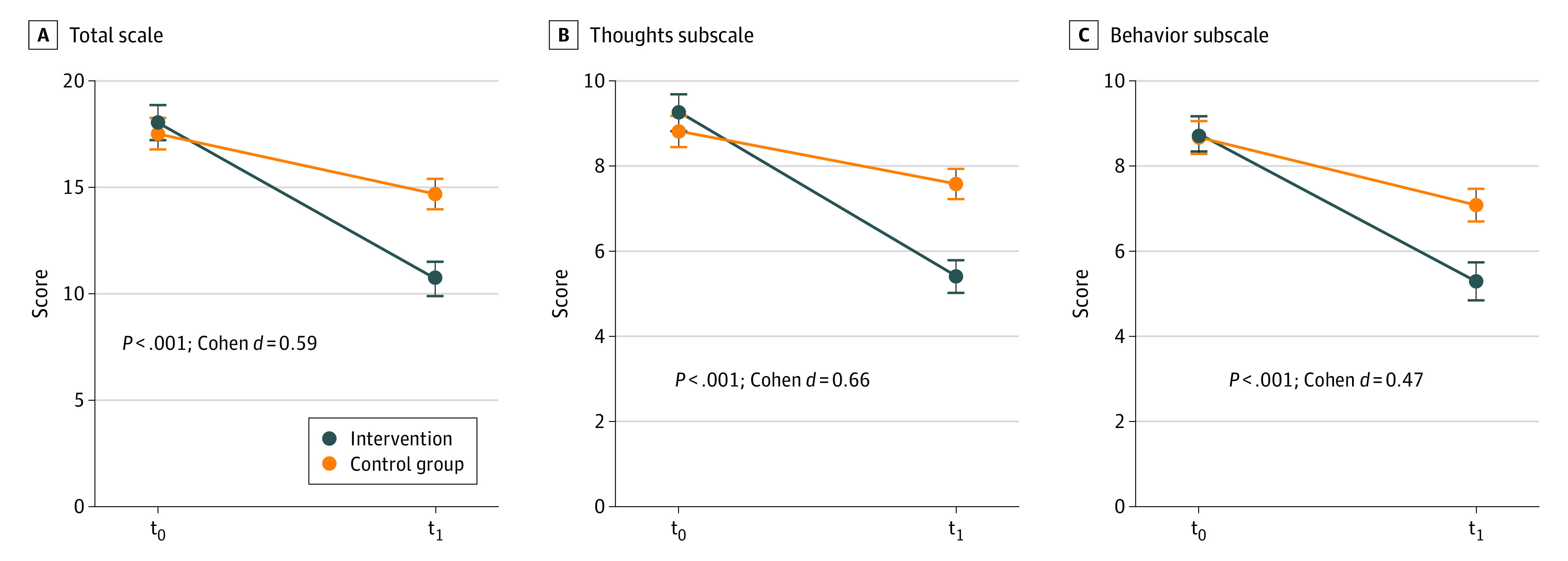
Pathological Gambling Adaptation of the Yale-Brown Obsessive-Compulsive Scale (PG-YBOCS) Scores t_0_ indicates before intervention; t_1_, 6 weeks after intervention.

Results of the intention-to-treat between-group analyses are depicted in Table 2 and Figure 2. The results indicate that the improvement of gambling symptoms (PG-YBOCS total scale [primary outcome]) was significantly greater in the intervention group than in the control group, with a mean difference of −3.35 (95% CI, −4.79 to −1.91; *P* < .001; Cohen *d* = 0.59). Between-group effects were also significant in favor of the intervention group for the subscales PG-YBOCS behavior (mean difference, −1.46; 95% CI, −2.25 to −0.67; *P* < .001; Cohen *d* = 0.47) and PG-YBOCS thoughts (mean difference, −1.85; 95% CI, −2.58 to −1.13; *P* < .001; Cohen *d* = 0.66) as well as for the secondary outcomes PHQ-9 (mean difference, −1.05; 95% CI, −1.87 to −0.22; *P* = .01; Cohen *d* = 0.33) and SOGS (mean difference, −1.46; 95% CI, −2.37 to −0.54; *P* = .002; Cohen *d* = 0.40) but not for GABS (mean difference, −1.62; 95% CI, −3.40 to 0.15; *P* = .07; Cohen *d* = 0.23).

**Table 2.** Mean Change From Baseline to Postassessment

Outcome	Intervention (n = 94)		Wait-listed control group (n = 97)		ANCOVA with respective baseline values as covariates (n = 243)		
	Mean change (95% CI)	Cohen *d* (95% CI)	Mean change (95% CI)	Cohen *d* (95% CI)	Mean difference (95% CI)	*P* value	Cohen *d*
PG-YBOCS total score	7.34 (5.80 to 8.91)	0.97 (0.72 to 1.21)	2.84 (1.43 to 4.24)	0.41 (0.20 to 0.61)	−3.35 (−4.79 to −1.91)	<.001	0.59
PG-YBOCS thoughts	3.89 (3.09 to 4.70)	0.99 (0.74 to 1.24)	1.25 (0.54 to 1.95)	0.36 (0.15 to 0.56)	−1.85 (−2.58 to −1.13)	<.001	0.66
PG-YBOCS behavior	3.46 (2.62 to 4.30)	0.84 (0.60 to 1.07)	1.59 (0.81 to 2.36)	0.41 (0.21 to 0.62)	−1.46 (−2.25 to −0.67)	<.001	0.47
PHQ-9	2.03 (1.03 to 3.03)	0.42 (0.21 to 0.63)	1.14 (0.25 to 2.04)	0.26 (0.06 to 0.46)	−1.05 (−1.87 to −0.22)	.01	0.33
GABS	5.00 (3.01 to 6.99)	0.52 (0.30 to 0.73)	2.36 (1.15 to 3.57)	0.39 (0.19 to 0.60)	−1.62 (−3.40 to 0.15)	.07	0.23
SOGS	4.43 (3.33 to 5.52)	0.83 (0.59 to 1.06)	2.68 (1.84 to 3.52)	0.64 (0.42 to 0.86)	−1.46 (−2.37 to −0.54)	.002	0.40

Abbreviations: ANCOVA, analysis of covariance; GABS, Gambling Attitudes and Beliefs Survey; PG-YBOCS, pathological gambling adaptation of Yale-Brown Obsessive Compulsive Scale; PHQ-9, Patient Health Questionnaire–9; SOGS, South Oaks Gambling Screen.

### Secondary Analyses

APOI and treatment expectations (PATHEV) for the 2 groups are presented in Table 1. The 2 groups did not differ on any of the (sub)scales. Considering a possible range of 16 to 80 for the total score of the APOI, a mean (SD) total score of 51.84 (7.61) for the whole sample can be regarded as a medium to high positive attitude toward online interventions. With a total score ranging from 11 to 55, a mean (SD) total score of 33.16 (4.93) of the whole sample for the PATHEV can be regarded as medium treatment expectations. No significant correlations were found between the 2 scales (APOI and PATHEV) and symptom change during the intervention period (difference scores from t_0_ to t_1_) for any of the outcomes or frequency of use.

Results of the exploratory moderation analysis are depicted in Table 3. Individuals without a depression diagnosis showed significant improvement on the primary outcome for the intervention group compared with the control group. Individuals in the intervention group who were employed full time (or who worked relatively more hours per week) showed significant improvement on the primary outcome compared with the control group. Furthermore, individuals in the intervention group who endorsed the statement that the use of an online intervention increases social isolation and loneliness (APOI item 15) and those who had a positive treatment expectation experienced significantly greater reductions on the primary outcome compared with the control group. Individuals in the intervention group who had more gambling-specific dysfunctional thoughts (GABS) and more severe gambling symptoms (SOGS) showed significant improvement compared with the control group.

**Table 3.** Moderators for Problematic Gambling Improvement (PG-YBOCS Total Difference Scores) in the Per-Protocol Sample

Outcome	β (SE) [95% CI]	*t*	*P* value	*P* value		
				−1 SD	0	+1 SD
Depression diagnosis	−6.61 (3.20) [−12.92 to −0.31]	−2.07	.04	<.001	NA	.83
Employment status	−1.69 (0.62) [−2.92 to −0.46]	−2.71	.007	<.001	<.001	.12
APOI item 15	2.22 (1.11) [0.04 to 4.41]	2.01	.046	.006	<.001	<.001
Treatment expectation^a^	4.28 (1.51) [1.30 to 7.27]	2.83	.005	.56	<.001	<.001
GABS total	0.33 (0.11) [0.11 to 0.55]	2.91	.004	.21	<.001	<.001
SOGS total	0.49 (0.22) [0.06 to 0.92]	2.25	.03	.07	<.001	<.001

Abbreviations: APOI, Attitudes Towards Psychological Online Interventions; GABS, Gambling Attitudes and Beliefs Scale; NA, not applicable; PG-YBOCS, pathological gambling adaptation of Yale-Brown Obsessive Compulsive Scale; SOGS, South Oaks Gambling Screen.

^a^ Treatment expectation was assessed with a global additional item (“I expect to feel much worse/somewhat worse/the same/somewhat better/much better after using the intervention”).

Results on subjective evaluation and acceptance are given in the eTable in Supplement 2. Overall, the intervention was positively evaluated; 69 participants (87.3%) rated the quality of the program as excellent or good, and 66 (83.5%) would (probably) recommend it to a friend with similar problems.

## Discussion

This study aimed to assess the acceptance and effectiveness of an enhanced self-guided internet-based intervention for problematic gambling behavior and to examine possible moderators of treatment outcome. As expected, the intervention significantly improved problematic gambling behavior and depressive symptoms compared with a wait-listed control group. Moderate effect sizes were observed for reducing gambling symptoms (Cohen *d* = 0.59), similar to the effect size reported in other studies (Hedges *g* = 0.47).^15^ We observed a small effect for reducing depressive symptoms (Cohen *d* = 0.33), whereas another study reported a slightly larger effect (Cohen *d* = 0.69).^16^ Similar to a prior study,^12^ we did not find significant effects on gambling-specific dysfunctional thoughts. No other study, to our knowledge, has yet explored the effect of an internet-based intervention on these thoughts.

Despite a high study completion rate of 78.6% compared with comparative studies with high attrition rates,^14,16,17^ treatment adherence was low, with a shorter mean (SD) use time of 61.22 (63.60) minutes compared with the previous study.^12^ However, participants completed more modules (mean [SD] number of modules completed, 4.55 [4.98]) possibly due to program modifications (shortened texts and modules as well as supplemental graphics). The intervention’s efficacy, despite its brief use, may be attributable to its focus on behavior change beyond the program by encouraging users to apply the exercises in their daily lives.

The improved effectiveness in the current study compared with the previous study^12^ may stem from significant enhancements to the intervention and the addition of a smartphone application. Furthermore, the larger sample enabled detection of even small effects, and the sample was less distressed by depressive symptoms at baseline compared with the previous study^12^ (mean [SD] PHQ-9 score, 8.33 [5.67] vs 11.08 [4.69]), and comorbid depressive symptoms are known to negatively affect treatment effects and adherence.^40^ This finding was further supported by the moderation analysis, which indicated greater benefits from the intervention for those without a depression diagnosis.

This study is the first, to our knowledge, to investigate attitudes toward internet-based interventions in individuals with problematic gambling behavior. Participants showed a moderately positive attitude, similar to findings in individuals with depression.^25,26,41^ Attitudes were not correlated or associated with treatment outcome, in contrast to a previous study.^41^ However, our sample’s positive attitude may be biased because we included only study participants.

Treatment expectations were moderate and did not correlate with treatment outcome and intervention use. However, expectations did moderate treatment outcome, with individuals in the intervention group who expected positive results benefiting more from the intervention compared with the control group, akin to placebo effects.^42^ Surprisingly, those anticipating increased isolation and loneliness through the online intervention also benefited significantly more from the intervention compared with the control group, possibly due to corrective experiences. Compared with another study,^18^ our assessment found lower expectations, probably because a different instrument was used.

### Limitations

The study has several limitations. Not mandating a formal diagnosis of a gambling disorder or a specific severity threshold for symptom severity may have widened the symptom severity range, potentially increasing the likelihood of a type I error. However, this approach aimed to increase the generalizability and thus the external validity of the study. In addition, this enabled more people to potentially benefit from the intervention. Limited resources precluded follow-up assessments, hindering conclusions on long-term effects. The short 6-week intervention period is another limitation.

## Conclusions

Within this randomized clinical trial, the effectiveness of the enhanced self-guided internet-based intervention was demonstrated in a sample of 243 individuals with self-reported problematic gambling behavior. Future studies should include follow-up assessments and active control groups to determine whether effects are sustained.

## Notes

Supplement 1.
Trial Protocol

Supplement 2. **eTable.** Subjective Appraisal of Restart Based on the German Version (ZUF-8) of the Client Satisfaction Questionnaire (CSQ-8), Frequencies and Percentages of Positive Answers

Supplement 3.
Data Sharing Statement
