# Supplementary material for: A Self-Guided Internet-Based Intervention for the Reduction of Gambling Symptoms: A Randomized Clinical Trial
Source: JAMA Netw Open. 2024 Jun 21;7(6):e2417282. doi: 10.1001/jamanetworkopen.2024.17282 (PMC11193125; doi:10.1001/jamanetworkopen.2024.17282)
Supplement: Supplement 1. — Trial Protocol [file jamanetwopen-e2417282-s001.pdf]

This document was originally submitted in German to the responsible ethics committee and was subsequently translated into English. Lara Bücker got married and is now named Lara Rolvien.

Title of the study: Further development of the computer-based training program "Restart" for individuals with gambling problems: A randomized controlled trial

Lead investigator: Dr. Lara Bücker
l.buecker@uke.de
University Medical Center Hamburg-Eppendorf
Center for Psychosocial Medicine
Clinical Neuropsychology Working Group
Martinistrasse 52
D-20246 Hamburg

Cooperation partner: Prof. Dr. Steffen Moritz
moritz@uke.de
University Medical Center Hamburg-Eppendorf
Center for Psychosocial Medicine
Clinical Neuropsychology Working Group
Martinistrasse 52
D-20246 Hamburg

Funding: No
Planned project period: April 2021 until April 2022

Background: According to a representative study of the 16- to 70-year-old population in Germany, 75.3% of respondents have participated in gambling at least once in their lives. The 12-month prevalence for problem gambling behavior is 0.39% and for pathological gambling behavior 0.34% (Banz, 2019). However, a high number of unreported cases is assumed here. The 12-month prevalence of sports betting in Germany was 2.2% in 2019 (Banz, 2019). Live sports betting on the internet is considered to have a high-risk potential (Meyer & Bachmann, 2017). Individuals with problematic or pathological gambling behavior are often very ashamed of their symptoms and have difficulty acknowledging them (Suurvali et al., 2012), which leads to reduced treatment uptake. Studies show that up to 90% of those affected do not seek treatment (Slutske et al., 2009). Internet-based interventions are one way of reducing the treatment gap for people with problematic and pathological gambling behavior (Bücker et al., 2019). These interventions are an effective alternative to conventional face-to-face interventions in the treatment of mental illness (Carlbring et al., 2018).

Objectives: The main objective of this study is to investigate the online program "Restart" (online self-help program for people with problematic gambling or gambling disorder) regarding its effectiveness, acceptance and participants' motivation for treatment. The basis is the version of the online program used in the preliminary study (Bücker, Westermann, Kuhn & Moritz, 2019; Bücker et al., under review) and the findings gained from it. In addition to an introductory module, the initial version of "Restart" included 10 further modules designed to help users overcome emotional and gambling-related problems. These are dedicated to the topics of "mindfulness", "positive activities", "sleep", "self-worth", "changing your thinking", "ABC scheme", "social skills", "dealing with gambling urges", "debt regulation" and "preventing relapses". In the new version to be examined, the online program was expanded to include a module on sports betting, an interactive module for building motivation (which

appears immediately after the first login to the program), reminders in the form of e-mails to increase adherence (twice a week) and a new layout (modernization of the user interface). A new smartphone app that can be used in combination is also offered.

Procedure: The planned study will be conducted as a randomized controlled trial. It is a two-arm study consisting of an intervention group, which will have access to the online program for 6 weeks after the baseline surveys, and a waitlist control group, which will have access to the intervention following the post-assessment. The baseline and post-surveys will assess socio-demographic and psychopathological and will be conducted online and anonymously via Questback®. The following instruments will be assessed: Sociodemographic data, Pathological Gambling Y-BOCS (PG-YBOCS), Patient Health Questionnaire PHQ-9), South Oaks Gambling Scale (SOGS), Gambling Attitudes and Beliefs Scale (GABS), Attitude Towards Psychological Online Interventions Questionnaire (APOI), Patient Questionnaire for Therapy Expectation and Evaluation (PATHEV), Positive and Negative Effects of Psychotherapy Scale for internet-based Interventions (PANEPS-I) and a questionnaire to record the subjective evaluation of the training (ZUF-8).

Online forums dealing with problematic gambling will be used for recruitment. In addition, a Google AdWords/Facebook campaign will be launched. Inclusion criteria for the study are age between 18 and 70 years, informed consent, internet access, sufficient command of the German language, willingness to participate in two anonymous online assessments each lasting approx. 25-30 minutes, willingness to participate in a 6-week online program, willingness to use the program at their own risk, presence of gambling symptoms, presence of a desire for treatment, and willingness to leave an e-mail address and receive e-mails twice a week. For the latter, instructions for creating a free and anonymous e-mail address are

provided. The presence of lifetime schizophrenic or bipolar disorder or acute suicidal tendencies are exclusion criteria.

Statistical analysis plan: The results will be analyzed using an intention-to-treat analysis (ITT) and a per-protocol analysis (PP). Missing values in the ITT will be checked using both LOCF (last observation carried forward) and multiple imputations (MI). An analysis of covariance (ANCOVA) with baseline values as covariates is used as the primary statistical analysis. The sample size calculation was performed with G*Power and resulted in a sample size of 199 subjects based on a small to medium effect size $f=0.20$ (based on the results obtained in the previous study), a power of .80 and an $\alpha=.05$. Considering an expected dropout rate of 20%, this results in a total sample size of 238 subjects.

Expected benefit: We expect a greater reduction in gambling symptoms in the intervention group compared to the wait-list control group. By modifying and optimizing the program, we expect that the effects of the first efficacy study can be increased (Bücker et al., under review). The Restart online program is constantly being improved and adapted to the needs of the target group. The study will also gain knowledge about attitudes towards online self-help programs among those affected by problematic gambling or gambling disorder and their motivation for treatment. Side effects were not assessed in previous, similar studies.
