# Supplementary material for: A Self-Guided Internet-Based Intervention for the Reduction of Gambling Symptoms: A Randomized Clinical Trial
Source: JAMA Netw Open. 2024 Jun 21;7(6):e2417282. doi: 10.1001/jamanetworkopen.2024.17282 (PMC11193125; doi:10.1001/jamanetworkopen.2024.17282)
Supplement: Supplement 2. — eTable. Subjective Appraisal of Restart Based on the German Version (ZUF-8) of the Client Satisfaction Questionnaire (CSQ-8), Frequencies and Percentages of Positive Answers [file jamanetwopen-e2417282-s002.pdf]

Supplementary Online Content

Rolvien LM, Buddeberg L, Gehlenborg J, Borsutzky SM, Moritz S. A self-guided internet-based intervention for the reduction of gambling symptoms: a randomized clinical trial. *JAMA Netw Open*. 2024;7(6):e2417282. doi:10.1001/jamanetworkopen.2024.17282

eTable. Subjective Appraisal of Restart Based on the German Version (ZUF-8) of the Client Satisfaction Questionnaire (CSQ-8), Frequencies and Percentages of Positive Answers

This supplementary material has been provided by the authors to give readers additional information about their work.

© 2024 Rolvien L et al. *JAMA Network Open.*

eTable. Subjective Appraisal of Restart Based on the German Version (ZUF-8) of the Client Satisfaction Questionnaire (CSQ-8), Frequencies and Percentages of Positive Answers

Item	No. (%)
How do you rate the quality of the program?*<br>(Excellent, good, not that good, bad)	69 (87.3)
Did you receive the type of treatment you expected to receive?*<br>(Absolutely, very much so, not really, not at all)	66 (83.5)
To what extent did the program meet your needs?*<br>(Very well, well, only partially, not at all)	49 (62.0)
Would you recommend the program to a friend with similar symptoms? # (Yes, probably, probably not, no)	66 (82.5)
How happy are you with the extent of the help you have received through using the program? #<br>(Very satisfied, mostly satisfied, somewhat dissatisfied, dissatisfied)	65 (81.3)
Did the program help you to cope with your problems more successfully?* (Absolutely, a lot, a little, not at all)	59 (74.7)
How satisfied are you with the program in general? #<br>(Very satisfied, mostly satisfied, somewhat unsatisfied, unsatisfied)	62 (77.5)
Would you use the program again?#<br>(Yes, probably, probably not, no)	63 (78.8)

Note. For each item, the first two responses (e.g., excellent, good) were deemed positive.
*n=79, #n=80
