# Supplementary material for: A Self-Guided Internet-Based Intervention for the Reduction of Gambling Symptoms: A Randomized Clinical Trial
Source: JAMA Netw Open. 2024 Jun 21;7(6):e2417282. doi: 10.1001/jamanetworkopen.2024.17282 (PMC11193125; doi:10.1001/jamanetworkopen.2024.17282)
Supplement: Supplement 3. — Data Sharing Statement [file jamanetwopen-e2417282-s003.pdf]

Data Sharing Statement

Rolvien. A Self-Guided Internet-Based Intervention for the Reduction of Gambling Symptoms. *JAMA Netw Open*. Published June 21, 2024. doi:10.1001/jamanetworkopen.2024.17282

Data
Data available: Yes
Data types: Deidentified participant data
How to access data: Data will be available upon request to the corresponding author.
When available: With publication

Supporting Documents
Document types: None

Additional Information
Who can access the data: Researchers whose proposed use of the data has been approved
Types of analyses: For a specified purpose, e.g. meta-analysis
Mechanisms of data availability: With a signed data access agreement
Any additional restrictions: None
